# Effects of Dietary Supplement of Basil Extract on Biochemical and Immunological Parameters and Growth Performance in *Oncorhynchus mykiss*

**DOI:** 10.1155/2024/5388049

**Published:** 2024-03-27

**Authors:** Mohammad Foad Noorbakhsh, Mehran Ghaemi, Amin Gholamhosseini, Amir Ali Heidari

**Affiliations:** ^1^Department of Basic Sciences, School of Veterinary Medicine, Shiraz University, Shiraz, Iran; ^2^Department of Pathobiology, School of Veterinary Medicine, Shiraz University, Shiraz, Iran; ^3^Department of Clinical Sciences, School of Veterinary Medicine, Shiraz University, Shiraz, Iran

## Abstract

*Ocimum basilicum* has antioxidant, anti-inflammatory, and antimicrobial effects. The present study was conducted to evaluate the effects of *O. basilicum* extract on growth yield, safety, and marinating physiologic functions of the rainbow trout. The fish were fed with food rations containing 0%, 1%, 2%, and 3% of basil extract. Basil extract, especially at 1% concentration food ration, significantly increased the growth parameters compared with the control group (*p* < 0.05). The biochemical parameters of hepatic function, renal function, glucose, triglyceride, and cholesterol level were significantly reduced in the treatment groups compared with the control group (*p* < 0.05). Feeding with basil extract led to a significant increase in Ig, lysozyme, and respiratory burst assay, with the most prominent elevation at 2% concentration food ration. The mucosal antibacterial activity was improved. The mortality rate after exposure to *Yersinia ruckeri* was lower in the treatment groups compared with the control group. The results of the present study suggest that adding 2% basil extract to the food rations of the fish may improve their physiologic function and growth yield and reinforce their immune system.

## 1. Introduction

Aquaculture is viewed as a sustainable approach to extend depleted fish populations and endangered fish types, preserve them, and close the gap between supply and demand for aquatic food [1–3]. Aquaculture operations productivity is affected by a number of interconnected aspects, including the aquatic environment, nutrition, and the farmed stock [4–6]. Maximizing these factors is the foundation of sustainable aquaculture [7]. In 2017, fish consumption accounted for 17% of animal protein intake and 7% of total protein intake in humans. In 2018, 46% of total fish production occurred through aquaculture, 62.5% of which belonged to inland aquaculture. 1.6% of the fish aquaculture belongs to rainbow trout [8]. This North American local fish has been cultivated due to compatibility with various weather conditions [9, 10]. Rainbow trout has attracted attention due to proper quality meat, easy cultivation, and entertainment [11]. Due to the increased need for rainbow trout, the pisciculture system has changed from extensive to intensive and semiintensive systems, increasing the risk of infectious diseases [12]. Weight gain, food yield, and disease prevention may be improved in the farmed fish by food additives [13]. Some additives such as hormones and antibiotics may cause adverse effects [14]. Using herbal supplements is among the new approaches leading to growth stimulation [15–18], reinforcing the immune system [19–21], and showed antimicrobial effects [22, 23]. The other advantage of using herbal products is that they are compatible with the environment, easily accessible, and have low prices [21]. Riveter and colleagues (2017) reported that *Lamiaceae* family are the most commonly investigated plants in aquaculture and include the highest number of species with immune-stimulant activity. Basil is a medium-sized member of this family, present in the tropical and subtropical regions of Asia, Africa, and South and Central America. This medicinal plant is widely used in traditional medicine for headaches, cough, intestinal worms, and as an antispasm agent [24]. The hydroalcoholic basil extract is rich in the polyphenol compounds such as flavonoid, flavonol, and tannins [25]; basil extract shows antioxidant activity and has been more effective than dexamethasone in the empirical controlling of inflammation of the lungs [26] and has been effective in preventing thioacetamide-induced renal damage [27]. Basil has immunomodulatory effects through increasing IL-1*β* and TNF*α* [28]. IL-1*β* has diverse physiological functions and its roles in regulating the inflammatory process are conserved in fish [29]. Moreover, basil extract shows remarkable antibacterial and antifungal effects against *Pseudomonas aeruginosa*, *Shigella* spp., *Listeria monocytogenes*, *Staphylococcus aureus*, *Fusarium* spp., and *Cryptococcus* spp. [30].

Considering the reported immune-boosting, antimicrobial, antifungal, and anti-inflammatory effects regarding basil in mammals, the present study is aimed to investigate the effects of this plant on the growth performance and immune system boosting in rainbow trout.

## 2. Materials and Methods

A total of 360 rainbow trout (*Oncorhynchus mykiss*) juveniles with a mean weight of 12.38 ± 10 g was purchased from the Dlakhan Propagation and Rearing Center (Fars province, Iran) and transferred to the School of Veterinary Medicine, Shiraz University. During the adjustment period, the fish were kept in 12,200-l freshwater tanks equipped with sponge filters (500 l h^−1^ of flow), for 2 weeks (30 fish in each tank), at the water temperature of 14.5 ± 1.1°C, dissolved oxygen of 8.1 ± 0.7 mg l^−1^, pH of 7.3 ± 0.4, electrical conduction of 693.83 ± 76 *µ*s/cm, and salinity of 0.3 ± 0.02 g l^−1^. The dark light period included 14 hr of light and 10 hr of darkness. A basic diet food ration was used during the adjustment period three times a day. Then, the fish were randomly divided into the four groups of control, and treatments 1, 2, and 3, each with three repetitions, and 0 (control), 1%, 2%, and 3% of basil extract were added to the basic food ration of the groups, respectively. The experimental procedures were reviewed and approved by the Ethics Committee for Animal Work at the School of Veterinary Medicine, Shiraz University. All procedure was according to the ethical guidelines for the care and use of live animals in experimental studies (IACUC. No. 4687/63).

### 2.1. Extracting

*Ocimum basilicum* was collected from Fars province in the Southwest of Iran and approved by the School of Agriculture, Shiraz University. The aerial parts of the plants were dried in a dark room under natural conditions, then ground into fine powder. The basil powder was mixed in a 1-l volumetric flask at 1 : 5 ratios with 80% methanol (one part of plant powder and five parts of 80% Methanol); the produced extract was filtered using Bucher Funnel and a paper filter. The solvent of the initial extract was evaporated using a rotary evaporator device for 4 hr at 40°C. The powder extract was maintained at 4°C until use [31].

### 2.2. Feeding with Experimental Food Ration

The basic feed was crushed, turned into a homogenous dough using distilled water, and mixed with basil extract at 0%, 1%, 2%, and 3% concentration food rations to prepare the experimental food ration. The resultant dough was then processed in the molding cylinder, dried at 45°C for 24 hr, spliced into 3-mm strings, and stored at 4°C. The fish were fed three times a day based on 3% body weight for eight weeks using plates containing 0%, 1%, 2%, and 3% basil extract for the control group, treatment groups 1, 2, and 3, respectively (Table 1).

### 2.3. Growth Yield

The samples were deprived of food for 24 hr before weighting and sampling, and the following parameters were measured at the end of the nutritional clinical trial.

  Weight gain (WG) = W2 (g)–W1 (g)
  Specific growth rate (SGR) = 100 (ln W2−ln W1) T^−1^
  Feed conversion ratio (FCR) = feed intake (g)/weight gain (g)
  Survival rate (SR) = (final amount of fish/initial number of fish) × 100
  where W1 represents initial weight, W2, final weight, and T time of trial period in days.

### 2.4. Sampling

Blood samples were taken from the posterior vein of each fish (12 fish from each experimental food ration, i.e., four fish of each tank) following anesthetization using clove oil (barijessence Co., 50 mg l^−1^, [31]). To provide adequate blood samples, the blood samples of each three fish with similar experimental food rations were piled up and immediately divided into two parts, one of which was transferred to the heparinized tube for hematology tests and respiratory burst assay. The other was transferred to the nonheparinized tube for immunologic and biochemical tests. The nonheparinized tube was centrifuged at 3,000x*g* for 15 min to separate serum, and stored at −80°C until use.

For mucus collection, the mucus was carefully removed from the upper surface of the body with a sterile spatula for scraping the fish from the front to the back. To get enough number of samples, six pools of four fish per treatment diet were sampled. The samples were entirely combined with the same amount of sterile Tris-buffered saline (TBS, 50 mM Tris–HCl, pH 8.0, 150 mM NaCl) for each sample and centrifuged (3,000x*g*, 4°C, 15 min) (Beckman Coulter, Avanti J−26 XPI). Then, the supernatants were filtered with Whatman No. 1 filter paper. To protect the filtrates against degradation and bacterial development, they were kept at −80°C until use.

### 2.5. Hematology

Following the Bolis method, the blood cell count was performed using Neubauer slide and Natt–Herrick dilution solvent. Hematocrit percentage (Ht%) was determined using the microhematocrit method. Hemoglobin level (Hb, g dl^−1^) was determined using the cyanmethemoglobin method by spectrophotometer device following the Drabkin's method. Moreover, leukocytes were counted using Giemsa-stained smears under a light microscope [32].

### 2.6. Serum Biochemical Parameters

Serum glucose, total protein, triglyceride, cholesterol, hepatic function enzymes including alanine aminotransferase (ALT), aspartate aminotransferase (AST), lactate dehydrogenase (LDH), and creatinine as the marker of renal health were measured using commercial kits (Pars Azmoun Company) by an autoanalyzer device (Model: Alpha-Classic Autoanalyzer, Tajhizat Sanjesh).

### 2.7. Mucus Bactericidal Activity

The bactericidal activity of skin mucosa against four pathogens including *Lactococcus garvieae* (EU727199), *Aeromonas hydrophila* (JF313402), *Yersinia ruckeri* (KC291153), and *Streptococcus iniae* (ATCC29178) were done *in vitro* using disk diffusion method [33]. Firstly, the pathogenic bacteria were cultivated in tryptic soy agar (TSA) at the temperature of 37°C for 24 hr. Then, 0.1 ml of the culture media (containing 1.5 × 10^6^ CFU ml^−1^ bacteria) was added to the Mueller Hinton agar. Then, the sterile paper disk (with a diameter of 5 mm) supplemented with 100 *µ*l of the skin mucous specimen was put over and incubated at the temperature of 25°C for 48 hr [34]. The measured diameter of the growth inhibition area was considered representative of the antibacterial capability of each sample.

### 2.8. Lysozyme Test

The lysozyme activity test of the blood samples was done based on the Ellis method with some modifications [35]. 25 *µ*L of serum was mixed with 1 ml of *Micrococcus lysodeikticus* suspension (0.2 mg ml^−1^ in a 0.05 M sodium phosphate buffer, pH 6.2), and the lysozyme activity was recorded using a spectrophotometer at the wavelength of 670 nm after 3 min and 30 s.

### 2.9. Immunoglobulin Content

Total plasma protein was measured using commercial kits (Pars Azmoon Co, Tehran, Iran), then Ig content was calculated as the difference between total protein content and albumin content.

### 2.10. Respiratory Burst Assay

Production of oxidative radicals by neutrophils during the respiratory burst was measured by Nitroblue Tetrazolium Test (NBT) based on Anderson and Siwicki's description [36]. Blood and NBT were mixed at a 1 : 1 ratio, and incubated at the temperature of 25°C for 30 min. Then 50 *µ*l of the mixture was put in a glass tube. To dissolve the reduced formazan product, 1 ml of dimethylformamide (Sigma, USA) was added and centrifuged at 2000x*g* for 5 min. Finally, the amount of reduced NBT of the supernatant fluid was measured in the wavelength of 540 nm. Dimethylformamide was used as the blank.

### 2.11. Quantitative Real-Time RT-PCR

Gene expression of *IL-1β* gene was measured by relative quantitative real-time RT-PCR method. Firstly, total RNA was extracted using the High Pure RNA Isolation Kit (Roche, Germany), according to the manufacturer`s instruction. The amount and purity of extracted RNAs were measured by a NanoDrop spectrophotometer. To ensure consistent results, we added only 0.5 *μ*g from each RNA sample to every real-time tube (Roche, Germany). Then, Taqman® probe real-time RT-PCR assay was conducted using One-step PrimeScript RT-PCR kit (Takara, Japan). Real-time RT-PCR reactions were performed in a 20 *μ*l total reaction volume comprised of 10 *μ*l 2x One-step RT-PCR Buffer III, 0.4 *μ*l TakaRa Ex Taq HS, 0.4 *μ*l PrimeScript RT enzyme Mix II, 0.4 *μ*l of each primer, and probe (10 *μ*M; Table 2), 1–5 *μ*l RNA templates (adjusted on 0.5 *μ*g), and 7.5–3.5 *μ*l RNase-free water. Reactions were carried out on a Light Cycler 96 (Roche, Germany) real-time PCR system under the following thermal program: 5 min of reverse transcription at 42°C and 1 cycle of 95°C for 10 s; followed by 40 cycles of denaturation at 95°C for 5 s, annealing and extension at 60°C for 20 s.

In the first run, the assay efficiencies of real-time RT-PCR assays were measured by five points of tenfold serial dilution of pooled RNA samples based on the slopes of the standard dilution curves. After optimum efficiencies were ensured, the main quantitative real-time RT-PCR assays were run on the RNA samples. Each treatment included three replicates, and each reaction was run in triplicate. *ELF1* was used as the normalizer gene and control group was selected as the calibrator. Finally, The Ct values of samples were analyzed by *ΔΔ*Ct method to measure relative gene expression.

### 2.12. Challenge Test

At the end of the 8^th^ week, the remaining fish undergo intraperitoneal injection of 0.1 ml of *Y. ruckeri* (KC291153) in PBS with a cellular density of 1.1 × 10^6^ cells/ml. 0.1 ml sterile PBS was injected as control. Previously, the challenge dose has been adjusted to cause 50% mortality (LD50) in no supplemented fish [37]. All fish were followed up for 2 weeks regarding clinical manifestations, abnormal behaviors, and mortality rate. Finally, RPC was evaluated [38].

### 2.13. Statistical Analysis

The Shapiro–Wilk test assessed the normality of all data. In order to run the statistical analysis, a one-way analysis of variance (ANOVA) was performed, followed by Duncan's multiple rangetests. Data were reported as, Mean ± SEM and the significance level was set at *p* < 0.05.

## 3. Results

### 3.1. Fish Growth

Adding 1% basil extract led to a significant increase (*p* < 0.05) in WG from 35 ± 0.46 g in the control group to 41.02 ± 0.47 g in the 1% treatment group, though 2% basil extract led to less substantial WG, which was not significant compared with the control. Moreover, 3% treatment group showed reduced WG compared with other groups, which showed a significant difference compared with 1% (*p* < 0.0001) and 2% (*p* < 0.01) treatment groups (Table 3). SGR demonstrated similar trends as WG (Table 3). Using a food ration containing 1% and 2% basil extract reduced FCR compared with the control group, though the difference was insignificant. Increased concentration of food ration of basil extract to 3%, significantly increased FCR compared with other groups (*p* < 0.05; Table 3). SR showed no significant difference between the groups (*p* > 0.05; Table 3).

### 3.2. Hematological Parameters

RBC count was increased in treatment groups 1%, 2%, and 3% compared with the control, which showed a significant difference in the 2% treatment group (Table 4, *p* < 0.01). WBC showed a similar trend, except that a significantly reduced WBC was observed in the 1% and 3% treatment groups compared with the 2% treatment group (Table 4, *p* < 0.01). Hematocrit was elevated in the treatment groups compared with the control group, which was significant in 2% treatment group (Table 3, *p* < 0.01). Hb was significantly improved in the 1% (*p* < 0.05) and 2% (*p* < 0.0001) treatment groups compared with the control group, with the highest increment reported in the 2% treatment group (Table 4). A significant reduction of Hb was observed in the 1% and 3% treatment groups compared with the 2% treatment group (*p* < 0.01). Circulating lymphocytes count was reduced in the treatment groups, and the most remarkable reduction was observed in the 2% treatment group (Table 4, *p* < 0.01). On the other hand, the neutrophil percentage was increased after treatment which was significant in the 2% treatment group (Table 4, *p* < 0.05). Monocyte and eosinophil percentage showed no significant difference (Table 4, *p* < 0.05).

### 3.3. Blood Biochemical Parameters

Glucose level was reduced in the treatment groups compared with the control, significantly in 2% group (Table 5, *p* < 0.0001). The creatinine level was significantly reduced in the treatment groups compared with the control group (*p* < 0.0001), the most remarkable reduction was observed in the 2% and 3% treatment groups, in which creatinine was significantly lower compared with the treatment group 1 (Table 5, *p* < 0.05). The serum ALT, AST, and ALP levels were reduced in the treatment groups (*p* < 0.0001); a significant reduction was observed in the 2% treatment group compared with 1% and 3% treatment groups (Table 5, *p* < 0.001). The serum LDH activity was reduced in the 1% and 2% treatment groups compared with the control group; the serum LDH level was significantly lower in the 2% treatment group compared with 1% treatment group (*p* < 0.0001), and the LDH level was elevated in the 3% treatment group compared with the control group (Table 5). Serum triglyceride and cholesterol were reduced in the treatment groups compared with the control group (*p* < 0.0001). The level of these factors was significantly reduced in the 3% treatment group compared with the other treatment groups (Table 5, *p* < 0.0001).

### 3.4. Blood Immunological Parameters

Serum total protein was significantly elevated in the 1% and 2% treatment groups compared with the control group (*p* < 0.0001), though it was reduced in 3% treatment group compared with the control group (Table 6, *p* < 0.0001). Serum total immunoglobulin alteration food ration trends were the same as the serum total protein (Table 6). Respiratory burst assay (RBA) of the treatment groups 1%, 2%, 3%, and the control group were 759.8 ± 9.17, 680.1 ± 14.73, 678 ± 17.47, 613.7 ± 7.34, respectively, and the treatment groups showed significant improvement compared with the control group (*p* < 0.01). The RBA was significantly elevated in the 2% treatment group compared with the other treatment groups (Table 6, *p* < 0.001). The highest serum lysozyme activities were observed in 2%, 1%, control, and 3% groups, respectively. The increased serum lysozyme activity in the 2% treatment group was significant compared with the control and 3% treatment group (Table 6, *p* < 0.05).

### 3.5. Antibacterial Effects of Skin Mucosa

**Figure 1:**
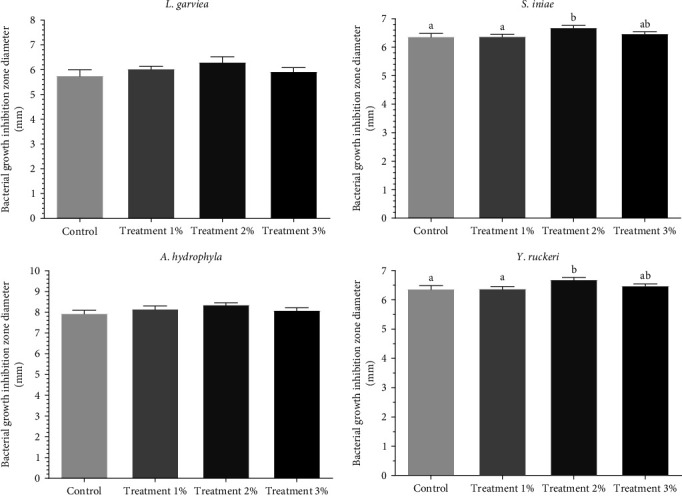
Effect of rainbow trout mucosa fed with different levels of *Ocimum basilicum* for 8 weeks on the diameter of the bacterial growth inhibition zone. Basil extract was added 0%, 1%, 2%, and 3% in control and treatment groups 1, 2, and 3, respectively. Results are presented as mean ± SEM. Different letters mean significant differences (*p* < 0.05).

The diameter of the bacterial growth inhibition zone increased in the treatment groups compared to the control group, but this difference was only significant for the *Y. ruckeri* and *S. iniae* bacteria between the 2% treatment group and the control group. Also, the antibacterial effect of mucosa in the 2% treatment group was significantly higher than 1% treatment group (*p* < 0.05, Figure 1).

### 3.6. Gene Expression

**Figure 2:**
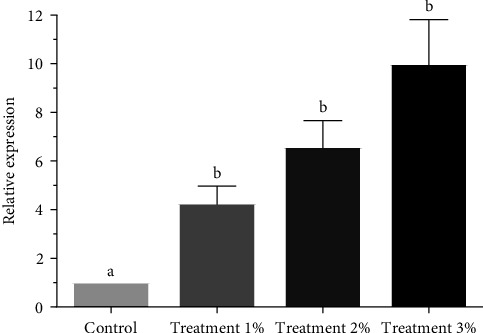
The expression ratio of *IL-1β* gene in different treatment groups. Results are presented as mean ± SEM. Different letters mean significant differences (*p* < 0.05).

The gene expression of *IL-1β* increased gradually from 1% to 3%. The expression of *IL-1β* gene in all groups was statistically significant (*p* < 0.05, Figure 2).

### 3.7. Challenge Test

**Figure 3:**
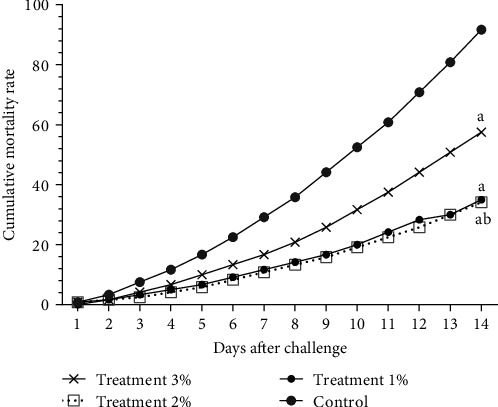
Challenge test *with Y. ruckeri* after 8 weeks of feeding with different concentrations of *Ocimum basilicum*. Basil extract was added 0%, 1%, 2%, and 3% in control and treatment groups 1, 2, and 3, respectively. Results are presented as mean ± SEM. Significant differences from the control and treatment 2% groups have been shown with a and b, respectively (*p* < 0.05).

At the end of the clinical trial, all groups were challenged with *Y. ruckeri*. The cumulative mortality showed reduced mortality in the treatment groups compared with the control groups (*p* < 0.001), and the lowest mortality was observed in the 2% treatment group (Figure 3).

## 4. Discussion

### 4.1. Fish Growth

In the present study, 1% and 2% basil extract improved growth parameters such as WG and SGR, which was significant with 1% basil extract, though 3% basil extract reduced these parameters even below the control group. Moreover, in the present study, FCR was reduced when using 1% and 2% basil extract compared with the control group, while it was increased using 3% basil extract. In 2008, El-Dakar and colleagues studied hybrid tilapia fish (*Oreochromis niloticus* x *O. aureus*), in which they used dried basil leaves as a nutrient absorber to improve the fish's appetite and hence increased the growth rate of tilapia fish. They showed that the addition of basil has increased the digestion of protein and preserved energy. Their study showed that a food ration containing 2% basil extract is more effective than 1%, which is not in line with the findings of the current study, since the present study showed the highest growth rate in the treatment group with 1% basil extract. This difference may be attributed to using different sources of basil; El-Dakar and colleagues used dried basil leaves while we used the methanolic extract of basil, and the palatability of the food ration containing 2%–3% of basil methanolic extract has declined [39]. Similar to the present study, Amirkhani and Firouzbakhsh' study (2015) [40] showed that basil leaf extract improved SGR and FCR, though it did not significantly affect SR [39]. The positive effects of basil addition to a fish diet may be attributed to the vital compounds such as essential fatty acids, minerals, and vitamins [41]. Moreover, in 2011, Daniel and colleagues showed that high concentration of Mg, Ca, K, and Na cations in basil. Additionally, basil increases the serum activity of amylase and lipase by improving pancreatic activity in fish [42].

### 4.2. Hematological Parameters

Hematological parameters play vital roles in determining animal welfare [43]. Using methanolic basil extract affected blood cells, and RBC and WBC increased, most prominently in the 2% treatment group. Hb and Ht% changes showed a similar pattern after using basil extract in the food ration. Although WBC, neutrophil, and lymphocyte counts increased and decreased significantly in the 2% treatment group, respectively, the monocytes and eosinophils percentages did not change significantly. In 2015, Amirkhani used ethanolic basil extract and improved RBC, WBC, Hb, and Ht%, which is completely consistent with the present study's findings [44]. The increase in WBC can be caused by the immune-stimulating effect of basil [28].

### 4.3. Biochemical Parameters

Liver function tests are crucial to assess liver physiology; thus, we used the serum activity of AST, ALT, and ALP in this study as bioindicators of fish liver changes [45, 46]. In the current study, the AST, ALT, and ALP enzyme activities were measured 8 weeks after adding basil extract to the food ration, which demonstrated a significant reduction compared with the control group. In 2021, a study on trout showed reduced AST and ALP levels after feeding with *A. Dracunculus* [31]. Additionally, basil extract decreased AST and ALP levels in rats with acetaminophen-induced liver damage [46]. LDH is a cytoplasmic biomarker enzyme playing an important role in the glycolytic cycle. LDH is present in all tissues, though the highest concentration of food rations belongs to the liver, skeletal muscle, and heart. In tissue damage, LDH activity increases depending on the severity and size of the cellular damage. LDH has always been highly important in fish [47]. In the present study, blood LDH levels were significantly reduced after basil treatment, owing to the basil's antioxidant effects. Basil contains phenolic and flavonoid compounds with antioxidant effects [48], preventing LDH release into the blood by preventing lipid peroxidation or improving cell membrane stability [27]. Reducing the level of LDH may also help fish immune systems against infection and stress [27].

The level of creatinine, a byproduct of muscle metabolism, increases as renal function decreases. Few nonrenal factors affect serum creatinine; hence, creatinine is a better renal function marker than BUN [49]. Basil has prevented the nephrotoxic effects of TAA and gentamicin in rats [27, 50] and acetaminophen in Balb/C rats [51], which is a sort of oxidative stress. Similarly, in the present study, basil could reduce creatinine levels as an indicator of renal function than the control group. Different concentrations of food rations of basil treatment reduced creatinine levels compared with the control group, which proved the normal renal function. Due to its antioxidant effects, basil can prevent lipids, proteins, and cellular DNA damage and maintain cell membrane and mitochondria integrity, and thus maintaining renal function [52].

Increased serum protein is a robust enduring parameter for monitoring the well-being of fish. In this study, the serum protein level was measured, which was elevated in the treatment groups with different concentrations of food rations of basil extract compared with the control group. Javed et al. [53] indicated that liver damage decreases serum protein; thus, improving liver function using a basil-containing food ration may have caused the increased serum protein in the treatment groups. On the other hand, the serum protein partly consists of synthetic humoral immunoglobulins [54], which were increased by using basil extract in the current study. Using dietary supplements has shown similar effects in various studies [55, 56].

Basil has shown antihyperglycemic effects in *in vitro* studies through *α*-amylase and *α*-glucosidase inhibition [57]. The hypoglycemic effects of basil have also been reported in diabetic rats [58]. Basil contains eugenol [59], which may inhibit *α*-glucosidase [60]. The basil hypoglycemic effects may be attributed to GLUT-4 [61]. In the present study, different concentrations of food rations of basil reduced serum glucose.

Basil leaf extract can reduce rat cholesterol levels for 1 week [62]. Polyphenolic compounds, particularly flavonoids can potentially reduce cholesterol levels [63]. Flavonoids and tannins decrease serum cholesterol levels through increased cholesterol metabolism to bile acids and increased cholesterol fecal excretion [64]. Another study showed the hypolipidemic effects of basil phenolic extract, containing remarkable amounts of rosmarinic acid, and decreased triglycerides and cholesterol in mice within a few hours [65]. In the current study, basil extract at 1%, 2%, and 3% concentration food rations decreased blood lipids compared with the control group. The most remarkable reduction in lipids was observed at the 3% food ration, which may be attributed to the combined increase in appetite and the effects of basil phenolic compounds.

### 4.4. Immunological Parameters

One of the major issues of fish farms is the spread of infectious diseases caused by pathogenic organisms leading to a great economic loss. Vaccines and antibiotics are commonly used to prevent the spread of infectious diseases, though they are costly and may elevate the risk of the emergence of antibiotic-resistant pathogens. Thus, using safe stimulants in aquaculture is becoming increasingly common [66]. Alcoholic basil extract modulates inflammatory factors such as TNF*α*, IL-1*β*, and IL-2 and suppresses iNOS induction and the NO product. This plant shows inhibitory effects on LOX and COX [67, 68]. Tsai et al. [69] showed the immunomodulatory effect of basil on human immune cells [69]. Another study in 2021 demonstrated the anti-inflammatory and immunomodulatory effects of basil through increasing IL-10 and TNF*α* levels [27]. In the current study, trout were fed with different consent food rations of basil to examine their anti-inflammatory and immunomodulatory effects. Studying the transcription of stress-, and immunity-related genes in the study of food additives on aquatic animals provides better gene-based interventions [70]. IL-1*β* is mainly produced by monocytes and macrophages and plays an important role in response to tissue damage [71, 72]. The expression of the *IL-1β* gene has been increased through feeding with different consent food rations of basil extract in a dose-dependent manner, demonstrating the regulation of the immune response in trout. The study on green tea showed similar results [73, 74]. Innate immunity is particularly important in evaluating the immune status of fish; thus, immunoglobulin, serum lysozyme activity, and RBA were studied as intrinsic humoral immune components [66]. The results of the present study indicated an improvement in innate humoral immunity. Increased lysozyme activity was reported as a component of hemorrhagic immunity compared to the control group, which is responsible for bacterial wall disruption [75].

Additionally, the present study showed that basil increased the mucosal activity against important pathogens in the aquaculture industry. The antimicrobial properties of basil have been demonstrated in various studies [50, 76]. Taecowisan et al. [77] indicated that the basil's antibacterial effect is related to the major components of basil extract, namely linalool and 1,8-cineol. Many studies have also shown the stimulatory effects of various herbal medicines, including *Lawsonia inermis* [78], oregano herb [74], and *Artemisia Drncuculus* [30], in boosting the immune system. The results of boosting the immune system and antibacterial activity of basil feeding at the end of the study were reflected in the reduced mortality rate in the challenge test.

## 5. Conclusions

This study showed that adding basil extract to the trout food improves their growth, boosts their immune system, improves their hepatic and renal function, and also decreases lipidemia and glucose of the rainbow trout. Other studies on fish farms also approved the present study findings in the laboratory conditions. According to the results of this study, adding 2% basil extract to the food ration of rainbow trout is recommended.

## Figures and Tables

**Table 1:** Dietary formulation of the experimental diet.

Ingredient	Amount (g/100 g ration)			
Corn flour	5.00	5.00	5.00	5.00
White wheat flour	5.00	5.00	5.00	5.00
Soya bean meal−48%	25.00	25.00	25.00	25.00
Fish oil	5.50	5.50	5.50	5.50
Fishmeal	45.00	45.00	45.00	45.00
Gelatin byproduct	2.00	2.00	2.00	2.00
Canola oil	5.00	5.00	5.00	5.00
Zeolite	4.00	4.00	4.00	4.00
Vitamin premix	1.50	1.50	1.50	1.50
Mineral premix	1.58	1.58	1.58	1.58
DL-Methionine	0.02	0.02	0.02	0.02
Coccidiostat	0.4	0.4	0.4	0.4
Herbal extract	0.00	1.00	2.00	3.00
Dry matter (%)	88.89	89.48	90.14	90.83
Metabolizable energy (Kcal g)	3.55	3.56	3.56	3.57
Protein (%)	44.83	44.91	44.98	45.04
Crude fat (%)	15.23	15.23	15.31	15.31
Crude fibre (%)	1.65	1.73	1.80	1.85
Total phosphorus (%)	1.31	1.31	1.31	1.31

**Table 2:** The sequences of primers and probes.

Primer/probe names	Sequence of primers and probes (5′-3′)	Target genes	PCR efficiencies
IL1b_For	CGAGTTCAAGGACAAGGA	*IL-1β*	1.97
IL1b_Rev	GACTCCAACTCCAACACTA	—	—
IL1b_Probe	FAM-TCTTCCACAGCACTCTCCAGC-BHQ1	—	—
ELF1_For	AGAGCTTCCAGGAATACC	*ELF1*	2.03
ELF1_Rev	CCTTGATGACACCAACAG	—	—
ELF1_Probe	FAM-CAGTCTGCCTCATGTCACGC-BHQ1	—	—

Primers and probes were designed in this study by Beacon Designer software (PREMIER Biosoft, USA).

**Table 3:** Growth yield of trout fed with different concentrations of *O. basilicum* for 8 weeks.

Group/parameters	Weight gain (g)	Specific growth rate (g/4 weeks)	Feed conversion rate	Survival rate (%)
Control	35.4 ± 0.46^a^	2.48 ± 0.04^a^	1.22 ± 0.01^a^	96.6 ± 3.4^a^
Treatment 1%	41.02 ± 0.47^b^	2.72 ± 0.03^b^	1.05 ± 0.01^a^	100 ± 0.0^a^
Treatment 2%	38.86 ± 2.04^b^	2.64 ± 0.08^b^	1.12 ± 0.06^a^	96.6 ± 3.4^a^
Treatment 3%	30.89 ± 1.71^ac^	2.72 ± 0.07^ac^	1.43 ± 0.08^b^	96.6 ± 3.4^a^

Data were presented in mean ± SEM. (*p* < 0.05). Different letters in each column show a significant difference (*p* < 0.05).

**Table 4:** Hematological parameters of trout fed with different concentrations of *O. basilicum* for 8 weeks.

Parameters/group	Control	Treatment 1%	Treatment 2%	Treatment 3%
RBC 106 ml^−1^	1.71 ± 0.032^a^	1.81 ± 0.028^a^	1.89 ± 0.031^b^	1.80 ± 0.027^a^
Hb (g dl^−1^)	6.39 ± 0.05^a^	6.55 ± 0.03^b^	6.74 ± 0.01^c^	6.48 ± 0.04^ac^
HtC (%)	38.3 ± 0.49^a^	40.60 ± 0.49^b^	42.5 ± 1.04^ab^	38.40 ± 0.68^ac^
WBC 103 ml^−1^	13.80 ± 0.11^a^	13.90 ± 0.07^b^	14.34 ± 0.15^a^	13.80 ± 0.07^ac^
Neutrophil (%)	16.57 ± 0.65^a^	18.80 ± 0.55^ab^	19.89 ± 0.91^b^	17.60 ± 0.78^ab^
Lymphocyte (%)	80.90 ± 0.55^a^	79.60 ± 0.63^b^	77.43 ± 0.97^ab^	80.60 ± 0.79^ac^
Monocyte (%)	1.30 ± 0.21^a^	1.30 ± 0.26^a^	1.50 ± 0.16^a^	1.55 ± 0.24^a^
Eosinophil (%)	0.20 ± 0.13^a^	0.30 ± 0.15^a^	0.40 ± 0.16^a^	0.20 ± 0.13^a^

Data were presented in mean ± SEM. Different letters in each row mean a significant difference (*p* < 0.05). RBC, red blood cell; Hb, hemoglobin; HtC, hematocrit; and WBC, white blood cell.

**Table 5:** Biochemical parameters of trout fed with different concentrations of *O. basilicum* for 8 weeks.

Parameters/group	Control	Treatment 1%	Treatment 2%	Treatment 3%
ALT (U dl^−1^)	0.75 ± 0.03^a^	0.44 ± 0.01^b^	0.23 ± 0.01^c^	0.62 ± 0.02^d^
AST (U dl^−1^)	359.3 ± 5.36^a^	178.30 ± 2.44^b^	142.40 ± 2.73^c^	227.70 ± 5.27^d^
ALP (U dl^−1^)	690.60 ± 2.40^a^	614.20 ± 3.14^b^	343.50 ± 7.22^c^	667.70 ± 10.08^ad^
LDH (U dl^−1^)	1684 ± 2.89^a^	2,596 ± 20.96^b^	1,055 ± 4.25^ac^	1738 ± 32.13^d^
Creatinine (mg dl^−1)^	0.76 ± 0.006^a^	0.45 ± 0.01^b^	0.37 ± 0.01^c^	0.40 ± 0.01^bd^
Glucose (mg dl^−1)^	58.90 ± 1.89^a^	54.10 ± 1.42^ab^	50.49 ± 0.26^b^	55.75 ± 2.68^ab^
Cholestrol (mg dl^−1^)	460.80 ± 2.62^a^	376.40 ± 4.43^b^	421.5 ± 5.16^c^	312.30 ± 6.52^d^
Triglyceride (mg dl^−1^)	802.1 ± 4.59^a^	588.70 ± 7.24^b^	595.00 ± 47.95^bc^	190.5 ± 11.41^d^

Data were presented in mean ± SEM. Different letters in each row mean a significant difference (*p* < 0.05). ALT, alanine transaminase; AST, aspartate transaminase; ALP, alkaline phosphatase; and LDH, lactate dehydrogenase.

**Table 6:** Immune stimulation of rainbow trout fed with different levels of *Ocimum basilicum* for 8 weeks.

Group/parameters	Total protein (g dl^−1^)	Total Ig (gdl^−1^)	Lysozyme activity	Respiratory burst assay
Control	3.38 ± 0.02^a^	2.29 ± 0.01^a^	1.90 ± 0.03^a^	613.7 ± 7.34^a^
Treatment 1%	3.91 ± 0.03^a^	2.30 ± 0.02^a^	1.98 ± 0.04^ab^	680.1 ± 14.73^b^
Treatment 2%	3.43 ± 0.02^a^	2.33 ± 0.01^a^	2.14 ± 0.04^b^	759.8 ± 9.17^c^
Treatment 3%	3.18 ± 0.03^b^	2.18 ± 0.01^b^	1.88 ± 0.03^ac^	678 ± 17.47^bd^

Data were presented in mean ± SEM. Different letters in each column mean a significant difference (*p* < 0.05).

## Data Availability

Data generated or analyzed during this study are available from the corresponding author upon reasonable request.
